# Bio-synthesis and characterization of silver nanoparticles from *Trichoderma* species against cassava root rot disease

**DOI:** 10.1038/s41598-024-60903-z

**Published:** 2024-05-31

**Authors:** Wannaporn Thepbandit, Narendra Kumar Papathoti, Nguyen Huy Hoang, Supatcharee Siriwong, Rungthip Sangpueak, Chanon Saengchan, Kansinee Laemchiab, Dusadee Kiddeejing, Kodchaphon Tonpho, Kumrai Buensanteai

**Affiliations:** 1 School of Crop Production Technology, Institute of Agricultural Technology, Suranaree University of Technology, Nakhon Ratchasima, 30000 Thailand; 2 Synchrotron Light Research Institute, Nakhon Ratchasima, 30000 Thailand

**Keywords:** Antifungal Plant Disease, Green Synthesis Nanoparticle, Cassava Root Rot disease, Lasiodiplodia theobromae, Fusarium solani, FTIR, Plant Biochemical, Silver nanoparticle, Biological techniques, Microbiology, Plant sciences, Ecology, Environmental social sciences

## Abstract

Cassava root rot disease caused by the fungal pathogens *Fusarium solani* and *Lasiodiplodia theobromae* produces severe damages on cassava production. This research was conducted to produce and assess silver nanoparticles (AgNPs) synthesized by *Trichoderma harzianum* for reducing root rot disease. The results revealed that using the supernatants of *T. harzianum* on a silver nitrate solution changed it to reddish color at 48 h, indicating the formation of AgNPs. Further characterization was identified using dynamic light scattering (DLS) and scanning electron microscope (SEM). DLS supported that the Z-average size is at 39.79 nm and the mean zeta potential is at − 36.5 mV. SEM revealed the formation of monodispersed spherical shape with a diameter between 60–75 nm. The antibacterial action of AgNPs as an antifungal agent was demonstrated by an observed decrease in the size of the fungal colonies using an increasing concentration of AgNPs until the complete inhibition growth of *L. theobromae* and *F. solani* at > 58 µg mL^−1^ and at ≥ 50 µg mL^−1^, respectively. At in vitro conditions, the applied AgNPs caused a decrease in the percentage of healthy aerial hyphae of *L. theobromae* (32.5%) and of *F. solani* (70.0%) compared to control (100%). The SR-FTIR spectra showed the highest peaks in the first region (3000–2800 cm^−1^) associated with lipids and fatty acids located at 2962, 2927, and 2854 cm^−1^ in the AgNPs treated samples. The second region (1700–1450 cm^−1^) consisting of proteins and peptides revealed the highest peaks at 1658, 1641, and 1548 cm^−1^ in the AgNPs treated samples. The third region (1300–900 cm^−1^), which involves nucleic acid, phospholipids, polysaccharides, and carbohydrates, revealed the highest peaks at 1155, 1079, and 1027 cm^−1^ in the readings from the untreated samples. Finally, the observed root rot severity on cassava roots treated with AgNPs (1.75 ± 0.50) was significantly lower than the control samples (5.00 ± 0.00).

## Introduction

Nanoparticles (NPs) are particles used in nanotechnology with a maximum size of 100 nm. The size, shape, and stability of nanoparticles are influenced by various factors, including the preparation technique, solvent properties, concentration, reducing agent strength, and temperature^1,2^. Scanning Electron Microscopy (SEM) and Dynamic Light Scattering (DLS) are techniques that can be used to determine the properties of nanoparticles, including their size and shape^3,4^. SEM is a highly effective technique for visualizing and characterizing individual nanoparticles that are deposited on a solid substrate^5^. On the other hand, DLS is better suited for the analysis of nanoparticles that are present in a liquid solution or suspension. It is common to employ a variety of methodologies in order to obtain a full comprehension of the characteristics of nanoparticles, including size, shape, and distribution^6,7^. NPs are in high demand due to their ability to link the gap between bulk elements and molecular composition^8^. In agricultural production, nanotechnology is widely used to develop products such as nanofertilizers, nanoherbicides, nanopesticides, and nanofungicides^9^. Such advances have gained a lot of popularity nowadays since it has contributed in boosting quality and crop yield production as well as pathogen reduction, seed vigor, photosynthesis, and the rate of nutrient uptake^10^. Moreover, the use of nanotechnology in agriculture systems have enhanced productivity while also restoring and increasing the ecosystem integrity through the reduction of chemical pollution and crop protection from environmental risks^11,12^. The conventional processes of synthetizing nanoparticles as a chemical technique were common due to the low energy requirements in the reduction process with producing homogeneous nanoparticles of high accuracy in size and form^13,14^. Although the process is more convenient, it can harm humans and the environment with its toxicity and residues^15^. Nevertheless, the bio synthesis of nano-scaled particles based on beneficial microbes is considered as an eco-friendly method. Microbial synthesis of nanoparticles can be performed in two ways: through microorganism biosurfactant and by using microorganism cells^16^. Biogenic silver nanoparticles (AgNPs) are antifungal or antibacterial agents that are ecologically friendly and safer than chemical-based and physical-based techniques^17^. *Trichoderma harzianum* secretes a large number of hydrolytic enzymes that act as reducing agents and stabilizer agents for the biosynthesis of nanoparticles^18,19^. Furthermore, *T. harzianum* can be a plant disease control agent which inhibits plant pathogens by means of producing a number of secondary compounds including protein-peptides, terpenoids, and other compounds as well as antibiotic enzymes^20^.

According to the International Potato Center, cassava root rot disease, caused by the fungal diseases *Lasiodiplodia theobromae* and *Fusarium solani*, has a severe negative impact on cassava production leading to yield losses of approximately 50%^21^. Several published studies suggested that AgNPs can control plant diseases and significantly delay mycelia growth of *Colletotrichum* sp., *Alternaria solani*, and *Botrytis cinerea*^22^. However, there is no research reported aiming to control cassava diseases. As a result, this study aimed to investigate the synthesis of biogenic AgNPs by *Trichoderma* isolates and to evaluate its efficacy on inhibiting root rot disease caused by *F. solani* and *L. theobromae* pathogens as a possible strategy for cassava disease control.

## Materials and methods

### Cultivation of microorganisms

The virulent fungal strains of *L. theobromae* and *F. solani* from the Plant Pathology and Biopesticide Laboratory (PPBL), Suranaree University of Technology, Thailand were cultured on potato dextrose agar (PDA) for seven and ten days, respectively. Conidia were harvested by gently scraping into sterilized water and the pathogen inoculum was prepared by adjusting the concentration to 1 × 10^6^ conidia mL^−1^ using a haemocytometer^23^.

*T. harzianum* and *T. virens* were cultivated individually in separated flasks containing potato dextrose broth (PDB) as medium at 30 ± 2 °C, and subjected to continuous shaking at 180 rpm for 48 hThen, the extracellular metabolites culture and the biomass were separated using centrifugation (Thermo Scientific, Germany) at 8500×*g* for 15 min^24^.

### Biogenic synthesis of silver nanoparticles

The preparation of AgNPs was conducted in two different conditions. For the first one, 5 g of biomass was transferred to 100 mL of 5 mM silver nitrate (AgNO_3_, analytical reagent grade, QReC, New Zealand) solution. For the second, 100 mL of extracellular metabolites culture was mixed with 100 mL of 5 mM AgNO_3_, then incubated in darkness until it experienced color change from light yellow to dark brown, which is an indication of the formation of AgNPs^7^. The AgNPs was separated using centrifugation at 8500 rpm for 15 min and rinsed with deionized water two times. Finally, the reactive mixture containing AgNPs was freeze-dried to completely remove the water contents^25^.

### Characterization of silver nanoparticles

#### Dynamic light scattering

The size and zeta potential of the AgNPs were measured by using a Zetasizer Nano ZS analyzer (Malvern Instruments Ltd., Worcestershire, UK). The AgNPs were diluted in water at 25 °C then loaded into the Zetasizer Nano ZS. The light from the laser emitter illuminated the sample in order to measure the dynamic fluctuations of light scattering intensity caused by the motion of the particles^26^.

#### Scanning electron microscope (SEM)

The surface morphology of the AgNPs was observed by using Scanning electron microscope (SEM) (JEOL JSM-6010LV, Japan). The AgNPs suspension was deposited on pin stubs^27^. The images of the size and morphology of AgNPs were recorded.

#### Field emission transmission electron microscopy (FE-TEM)/scanning transmission electron microscopy-energy dispersive X-ray spectroscopy (STEM-EDS) analysis

To determine the size, shape, and elemental composition of synthesized nanoparticles, 10 µL of the sample was applied onto on copper grids coated with formvar/carbon and allowed to dry overnight, then subjected to FE-TEM/STEM-EDS (Thermos Scientific TALOS F200X, USA) at 200 kV. The FE-TEM was used to capture high-resolution images of the AgNPs and STEM-EDS was used to obtain compositional information.

### Determination of inhibition zone by agar plate assay

Four different concentrations of bio-synthetized AgNPs at 20, 30, 40, and 50 µg mL^−1^ were used along with two positive controls (AgNO_3_ at 100 µg mL^−1^, and carbendazim at 4 mg mL^−1^) and one negative control (water). The antimicrobial activity was determined by the agar plate assay^28^. 100 µL of each previously described solutions were spread on a PDA agar plate; then, an 8-mm-agar disc of the mycelium of pathogen was deposited on each plate center and incubated at 25 ± 2 °C. The fungal mycelium was measured at 7 days post incubation. The results were recorded as a percentage value correlated to mycelium growth inhibition using the following simple equation:

$$\text{Inhibition Rate }\left({\%}\right)= \frac{{\text{A}}-{\text{B}}}{{\text{A}}} \times 100$$

where A and B were the maximum and the minimum values of the mycelium, respectively.

### Minimum inhibitory concentration (MIC) assay

The MIC of the AgNPs was determined in aseptic 96-well plates. One hundred microliters of spore suspension containing each *F. solani* and *L. theobromae* with a density of 1 × 10^5^ spores mL^−1^ in PDB was added to 100 μL of PDB containing the AgNPs in different final concentrations from 40 to 60 µg mL^−1^. The spore suspension was stored at 25 ± 2 °C for 72 h in the E24 incubator (New Brunswick, Germany). The inhibition of growth was determined by measuring the light absorbance of the mixture at 750 nm by using the Epoch micro-plate spectrophotometer (Biotek, Germany)^29,30^.

### In vitro culture of *Lasiodiplodia theobromae* and *Fusarium solani* with AgNPs

In order to test the toxicity of AgNPs, the samples were introduced into the PDA in three different concentrations (0, 40, and 50 µg mL^−1^). Two milliliters of *L. theobromae* and *F. solani* spore suspension (1 × 10^5^ spores mL^−1^) were amended onto the medium and incubated at 28 ± 2 °C for 12 h under fluorescent light and 12 h under darkness. Subsequently, the aerial hyphae growth was observed under light microscopy.

### Biochemical changes of fungi mycelium by Synchrotron radiation-based Fourier-transform infrared (SR-FTIR) microspectroscopy

The biochemical changes of the pathogens mycelium *L. theobromae* and *F. solani* treated with bio-synthetized AgNPs as well as the positive and negative control samples were analyzed by using SR-FTIR microspectroscopy. The *F. solani* growth on PDA was incubated at 28 ± 2 °C for ten days and the *L. theobromae* on PDA was incubated at 28 ± 2 °C for seven days. Later, a 8 mm diameter cork borer was used to drill at the edges of the pathogen colonies and transfer the samples to the PDB amended with two concentrations of biosynthesized AgNPs (40 and 50 µg mL^−1^), AgNO_3_ as a positive control (50 µg mL^−1^), and untreated. Next, the pathogen mycelium was incubated at 28 ± 2 °C for 72 h. The mycelium were collected by centrifugation (Thermo Scientific, Germany) at 8500×*g*, 4 °C for 15 min, and then washed twice with nuclease free water. A mass of mycelium was placed into barium fluoride windows (BaF_2_)^31^. The mycelium were dehydrated in vacuum for 24 h; then, subjected to SR-FTIR microspectroscopy. The spectral data of each sample was collected with a resolution of 6 cm^−1^ and a scan time of 64 at the beamline 4.1 Infrared Spectroscopy and Imaging, the Synchrotron Light Research Institute (SLRI), Thailand. The obtained spectral data were analyzed using the OPUS 7.5 software (Bruker Ltd., Germany). Using the Unscrambler X 10.5 software, the original spectra were converted to second derivatives and vector normalized unity for multivariate statistical analysis. This experiment was performed in four replications and repeated three times.

### Effect of bio-synthesis AgNPs to control cassava root rot disease

In order to determine the potential of biosynthesized AgNPs on limiting *L. theobromae* and *F. solani*, the adopted evaluation procedure was slightly adapted from Onyeka et al.^32^ and Boas et al.^33^. Whole cassava root cv. CMR 89 from the Department of Agriculture, Ministry of Agriculture and Cooperatives, NakhonRatchasima, Thailand was sterilized with 1% of sodium hypochlorite (NaClO) solution and washed twice with distilled water. Then, an 8 mm of cork borer was used to make uniform holes of 4 mm in depth of the root. The root samples of each treatment were sprayed with 10 mL of bio-synthetized AgNPs, positive control, and negative control, then left until dry in a laminar flow. Afterwards, 100 µL of fungal spore suspension with density of 1 × 10^5^ spores mL^−1^ was dropped on the drilling points on the roots^32,33^. The roots were kept in a container containing moistened cotton; then, incubated for 10 days. The disease symptoms appearing on the tested root samples were analyzed and rated based on a scale of disease severity for the cassava root rot disease, specified as follows: 1 = no mycelia formation, 2 = 1–25% mycelia formation, 3 = 26–50% mycelia formation, 4 = 51–75% mycelia formation, and 5 = mycelia covering the surface of the slice.

### Statistical analysis

All experiments were repeated four times, and the average data for each experiment and replication were reported. After being analyzed the data, an analysis of variance (ANOVA) was performed on it (SPSS software, version 19). The magnitude of the F value (P = 0.05) was used to assess the significance of the different treatments. Duncan's Multiple Range Test (DMRT) was used to differentiate between mean differences.

### Statement of permissions and/or licenses for collection of plant or seed specimens

The authors declare that the cassava root specimens used in this study are publicly accessible cassava cultivar and we were given explicit written permission to use them for this research.

### Plant guidelines

Experimental research and field studies on plants, including the collection of plant material comply with relevant institutional, national and international guidelines and legislation—Formal ethical approval is not required.

## Results

### Bio-synthesis of silver nanoparticles

**Figure 1:**
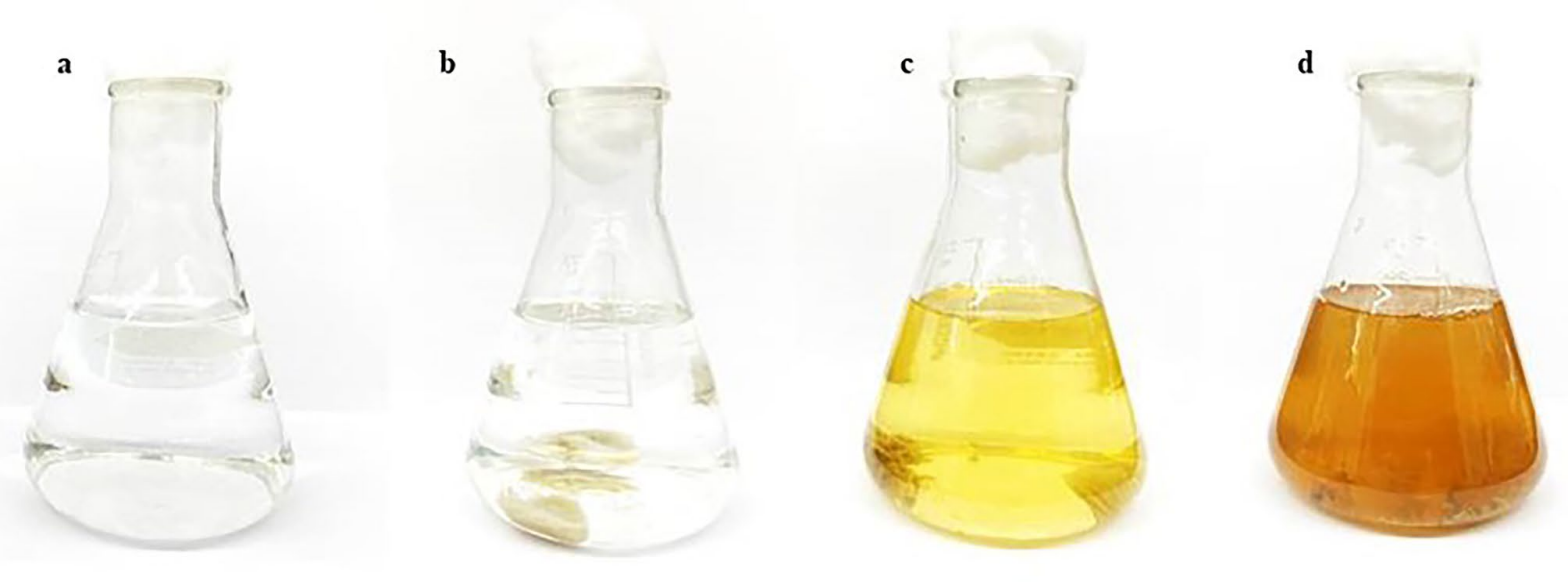
Color changes during the biosynthesis of AgNPs from 5 mM of silver nitrate solution with 5 g of *T. harzianum* biomass at different reaction times: (**a**) silver nitrate solution without *T. harzianum* biomass, (**b**) biosynthesis of AgNPs at 0 h, (**c**) biosynthesis of AgNPs at 24 h, and (**d**) biosynthesis of AgNPs at 48 h.

**Figure 2:**
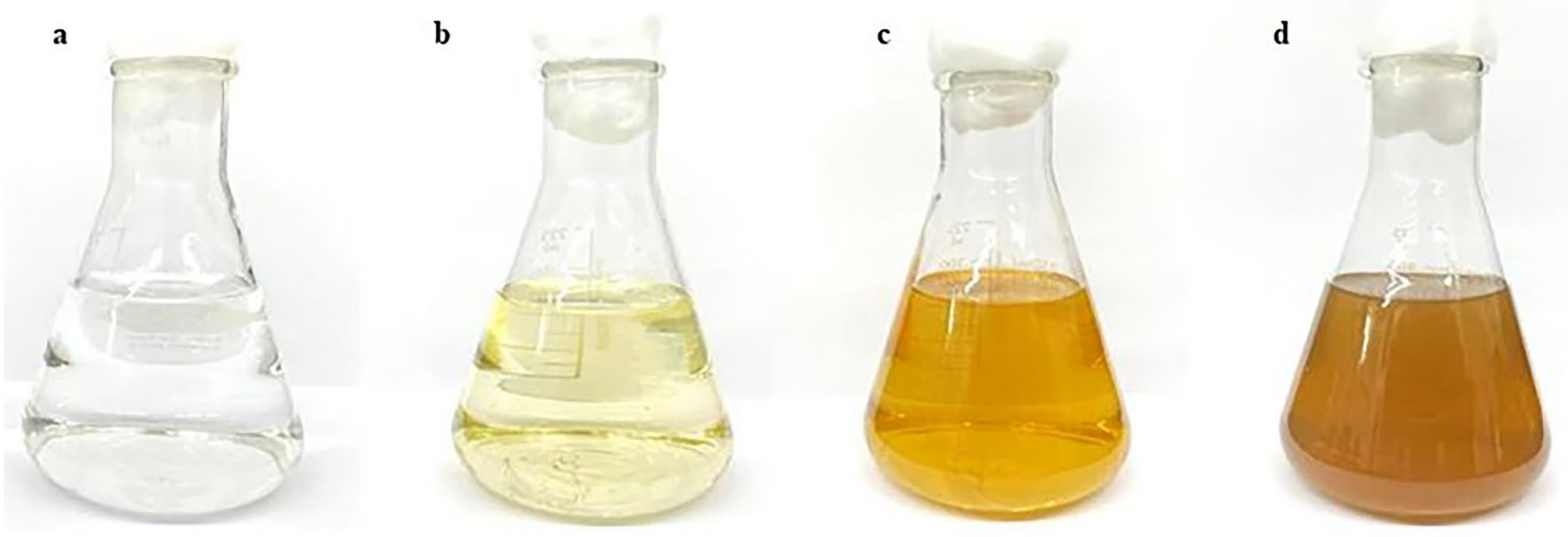
Color changes during the biosynthesis of AgNPs from 5 mM of silver nitrate solution with *T. harzianum* supernatant at different reaction times: (**a**) silver nitrate solution without *T. harzianum* supernatant, (**b**) biosynthesis of AgNPs at 0 h, (**c**) biosynthesis of AgNPs at 24 h, and (**d**) biosynthesis of AgNPs at 48 h.

In this study, two different strains of Trichoderma species, *T. harzianum* and *T. virens*, were screened for the synthesis of AgNPs. Results showed that the biosynthesis of AgNPs was accomplished by combining 100 mL of 5 mM AgNO_3_ solution with 5 g of Trichoderma species biomass or 100 mL of its supernatant. The implementation of this methodology produced the synthesis of AgNPs. The synthetization of AgNPs was confirmed by a color change from transparent to yellow and finally to reddish (Figs. 1 and 2) as was similarly observed when synthetizing AgNPs from *T. virens.*

### Characterization of silver nanoparticles

#### Dynamic light scattering

The results obtained from the DLS showed a mean size of 64.92 and 41.78 nm, and a zeta potential of − 38.9 and + 39.14 mV for the AgNPs synthesized using *T. virens* biomass and supernatant, respectively. Whereas, 59.2 and 39.79 nm were recorded from AgNPs samples synthesized using *T. harzianum* biomass and supernatant, respectively; and mean zeta potentials of − 23.7 and − 36.5 mV, respectively (Table 1). The synthesized AgNPs with the smallest recorded average particle size was used for further analysis.

**Table 1:** The size (nm) and zeta potential (mV) of biosynthesis AgNPs.

Treatment	Average particle size (nm)*	Average zeta potential (mV)*
AgNPs synthesized using *T. virens* biomass	64.92 ± 0.5	− 38.9 ± 0.2
AgNPs synthesized using *T. harzianum* biomass	59.2 ± 0.7	− 23.7 ± 0.1
AgNPs synthesized using *T. virens* supernatant	41.78 ± 0.8	+ 39.14 ± 0.3
AgNPs synthesized using *T. harzianum* supernatant	39.79 ± 0.5	− 36.5 ± 0.3

Data are shown as ± mean standard deviation of four replications.

#### Scanning electron microscope analysis

**Figure 3:**
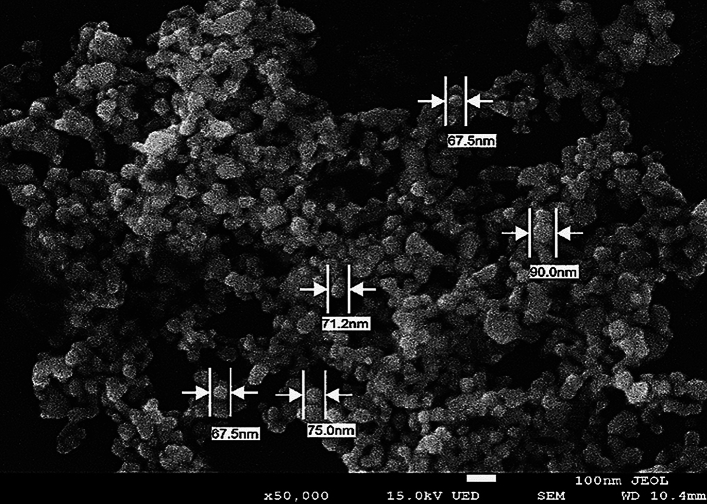
Scanning electron microscope (SEM) image of silver nanoparticles synthesized by *Trichoderma harzianum* supernatant from silver nitrate.

The morphology of the AgNPs synthesized using *T. harzianum* supernatant was further analyzed using SEM. The SEM image clearly showed the synthesis of particles which measured 60–75 nm (Fig. 3), confirming the development of silver nanostructures.

#### FE-TEM/STEM-EDS analysis

**Figure 4:**
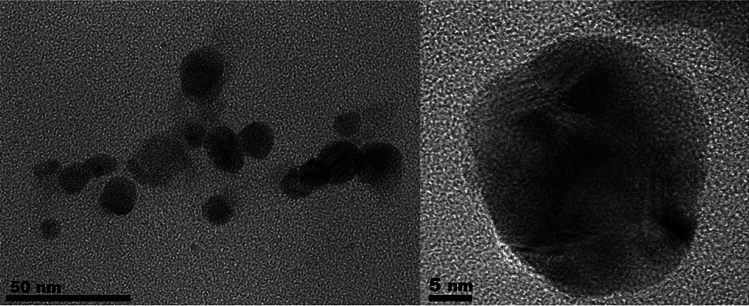
High resolution transmission electron microscopy (HR-TEM) image of silver nanoparticles synthesis by *Trichoderma harzianum* supernatant.

**Figure 5:**
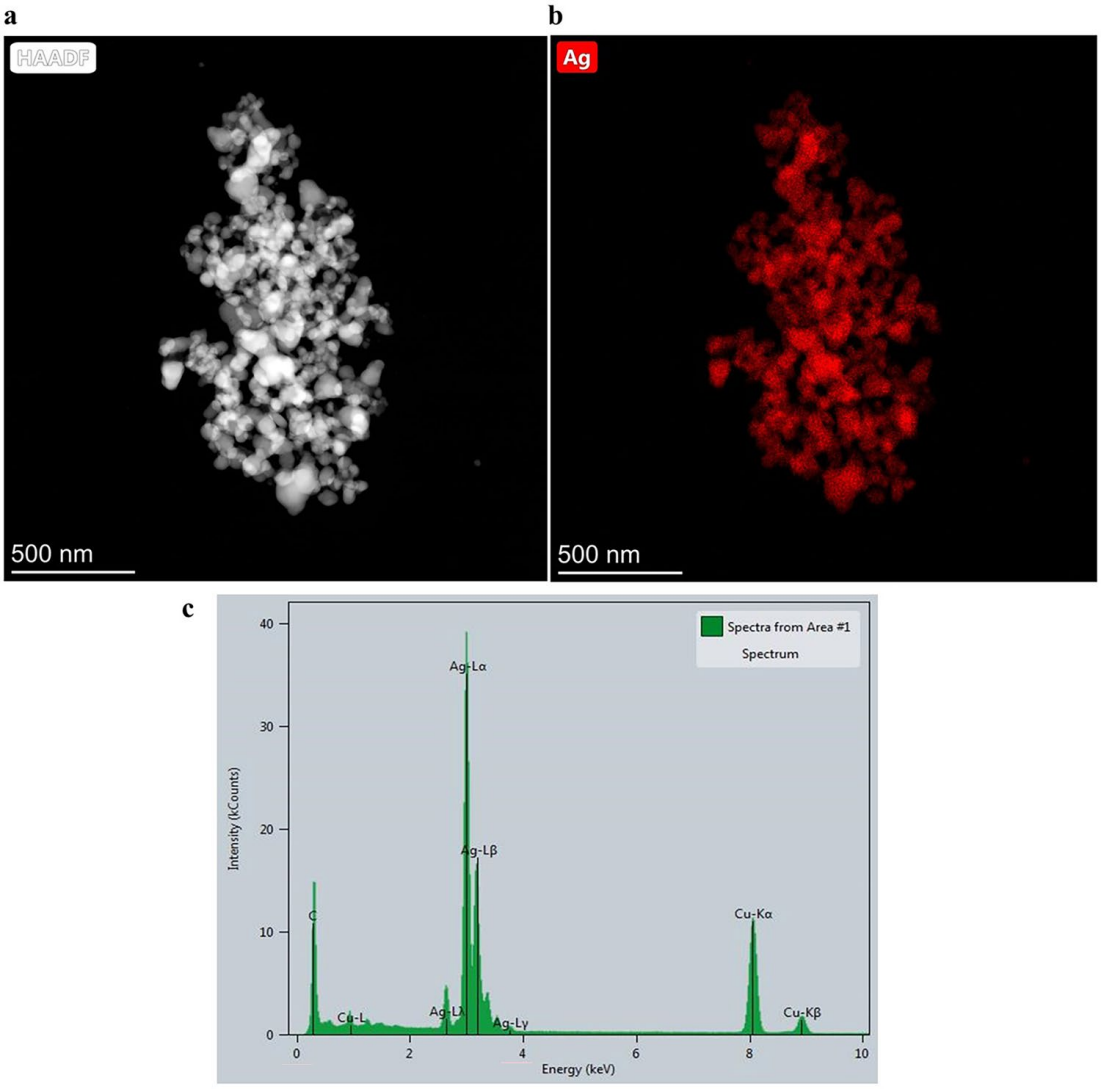
Scanning transmission electron microscopy (STEM)/energy dispersive X-ray (EDS). Mapping of silver nanoparticles (AgNPs) synthesis by *Trichoderma harzianum* supernatant; (**a**) TEM image of AgNPs using the HAADF detector, (**b**) mapping of silver (Ag) element, and (**c**) EDS spectrum of distribution in AgNPs.

A comprehensive structural investigation of AgNPs was conducted using high-resolution transmission electron microscopy (HR-TEM). The HR image of the AgNP crystal structure displayed nanoparticles that were uniformly spherical in shape and had an estimated size of less than 50 nm (Fig. 4). EDS was performed to confirm the elemental distribution in AgNPs. The HAADF image and EDS mapping of AgNPs revealed a strong signal for Ag and was mainly localized in the core domain of AgNPs (red) (Fig. 5a,b). The EDS spectrum of nanoparticles showed major peak at 2.9846 keV for silver metal. The other metal ions, including carbon (C) and copper (Cu), also appeared in the EDS spectrum, which can detect X-rays generated from the substrate material of the TEM grid.

### Determination of the antimicrobial activity and minimum inhibitory concentration

**Figure 6:**
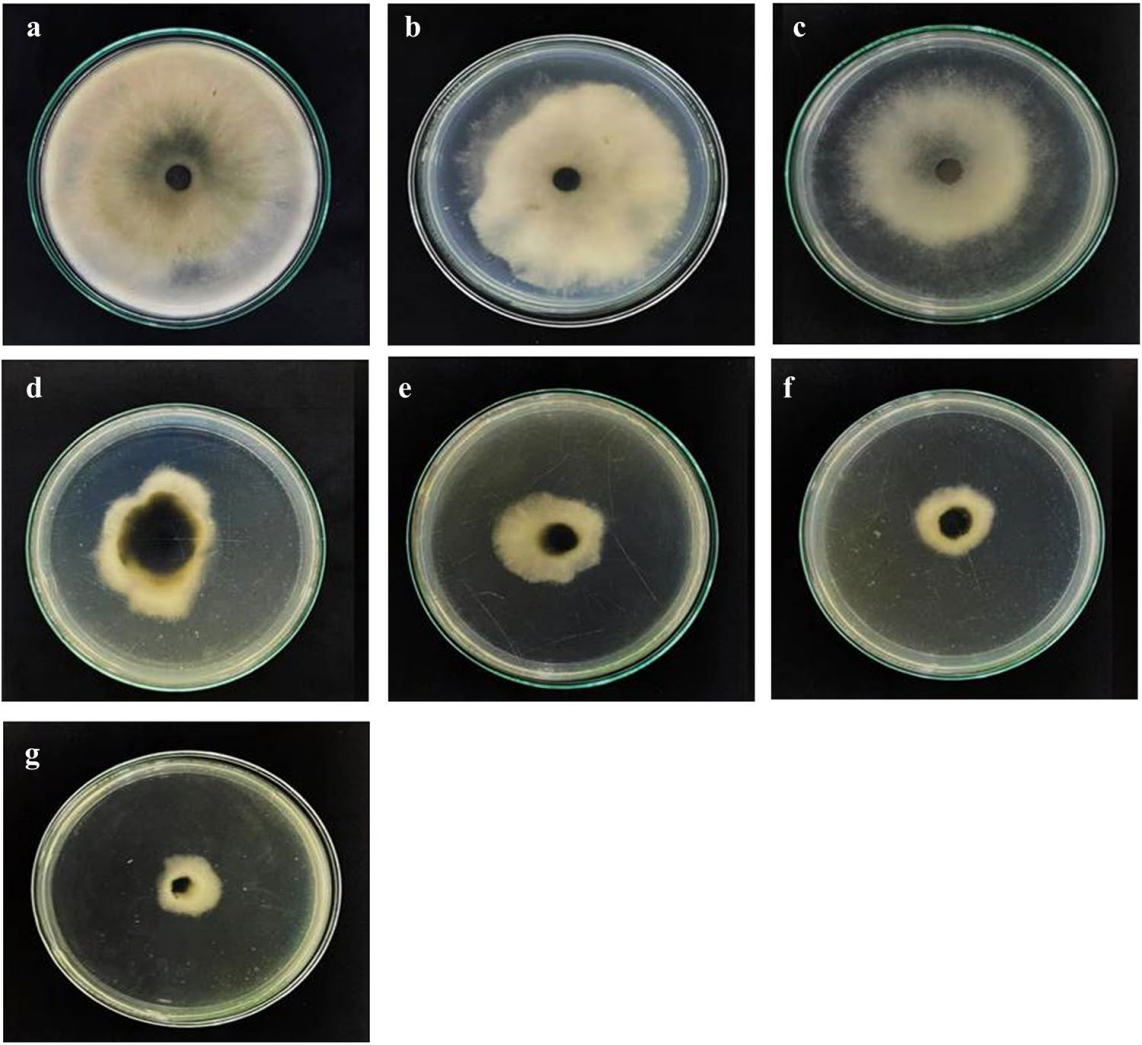
The antimicrobial activity of the silver nanoparticles (AgNPs) biosynthesized by *Trichoderma harzianum* inhibited *Lasiodiplodia theobromae* growth on potato dextrose agar plates. The plates were supplemented with varying concentrations of AgNPs as follows: (**a**) 0 µg mL^−1^, (**b**) 20 µg mL^−1^, (**c**) 30 µg mL^−1^, (**d**) 40 µg mL^−1^, (**e**) 50 µg mL^−1^, (**f**) 100 µg mL^−1^ silver nitrate, and (**g**) 4 mg mL^−1^ carbendazim.

**Figure 7:**
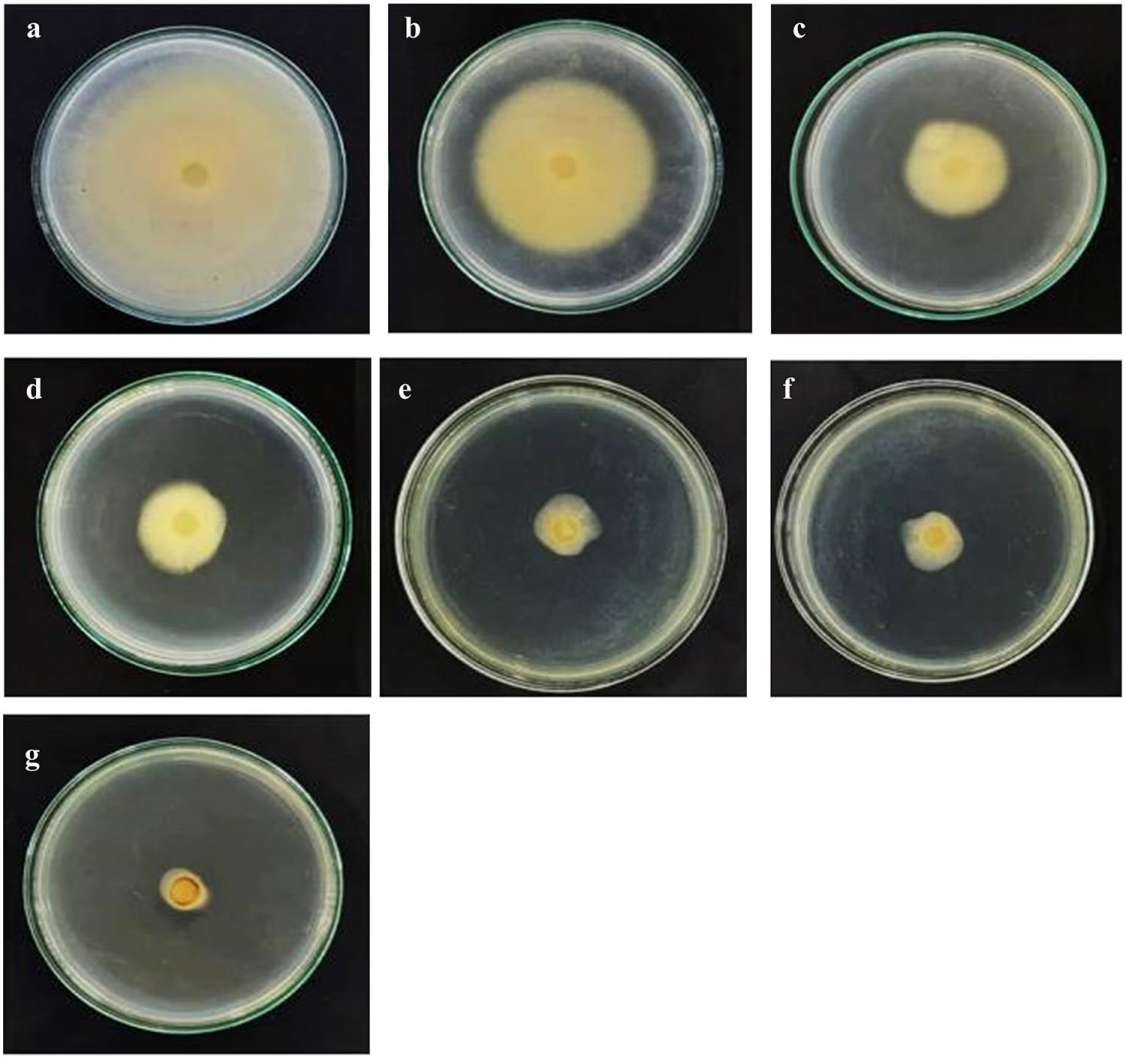
The antimicrobial activity of the silver nanoparticles (AgNPs) biosynthesized by *Trichoderma harzianum* inhibited *Fusarium solani* growth on potato dextrose agar plates. The plates were supplemented with varying concentrations of AgNPs as follows: of AgNPs: (**a**) 0 µg mL^−1^, (**b**) 20 µg mL^−1^, (**c**) 30 µg mL^−1^, (**d**) 40 µg mL^−1^, (**e**) 50 µg mL^−1^, (**f**) 100 µg mL^−1^ silver nitrate, and (**g**) 4 mg mL^−1^ carbendazim.

The antimicrobial activity of biosynthesized AgNPs against *L. theobromae* and *F. solani* was compared to the positive control (silver nitrate, carbendazim) and the negative control (water). The results indicated that the size of the fungi colonies decreased when increasing the concentration of AgNPs (Figs. 6, 7) until completely inhibiting the growth of *L. theobromae* at ≥ 58 µg mL^−1^ and *F. solani* at AgNPs ≥ 50 µg mL^−1^. Meanwhile, AgNO_3_ at an equal or larger dose than 120 µg mL^−1^ completely inhibited the growth of *L. theobromae* and *F. solani* (Table 2).

**Table 2:** Inhibition rate and minimum inhibitory concentration (MIC) at different concentrations of the AgNPs against *Lasiodiplodia theobromae and Fusarium solani* causing cassava root rot disease.

Pathogens	Zone of inhibition (%)						
	AgNPs (µg mL^−1^)					AgNO_3_ 100 µg mL^−1^	Carbendazim 4 mg mL^−1^
	0	20	30	40	50		
*L. theobromae*	0 ± 0^f^	12.80 ± 2.87^e^	30.05 ± 4.66^d^	54.66 ± 4.65^c^	79.72 ± 0.58^b^	83.98 ± 0.83^ab^	87.37 ± 0.76^a^
*F. solani*	0 ± 0^f^	20.22 ± 3.77^e^	66.61 ± 1.55^d^	76.22 ± 0.96^c^	83.66 ± 1.12^b^	85.88 ± 1.68^ab^	89.58 ± 0.53^a^
*L. theobromae* MIC	58 µg mL^−1^					120 µg mL^−1^	5 mg mL^−1^
*F. solani* MIC	55 µg mL^−1^					120 µg mL^−1^	4 mg mL^−1^

The meaning of the different letters (a, b, c) indicated significant difference via DMRT at P = 0.05.

### In vitro culture of *Lasiodiplodia theobromae* and* Fusarium solani* with AgNPs

**Figure 8:**
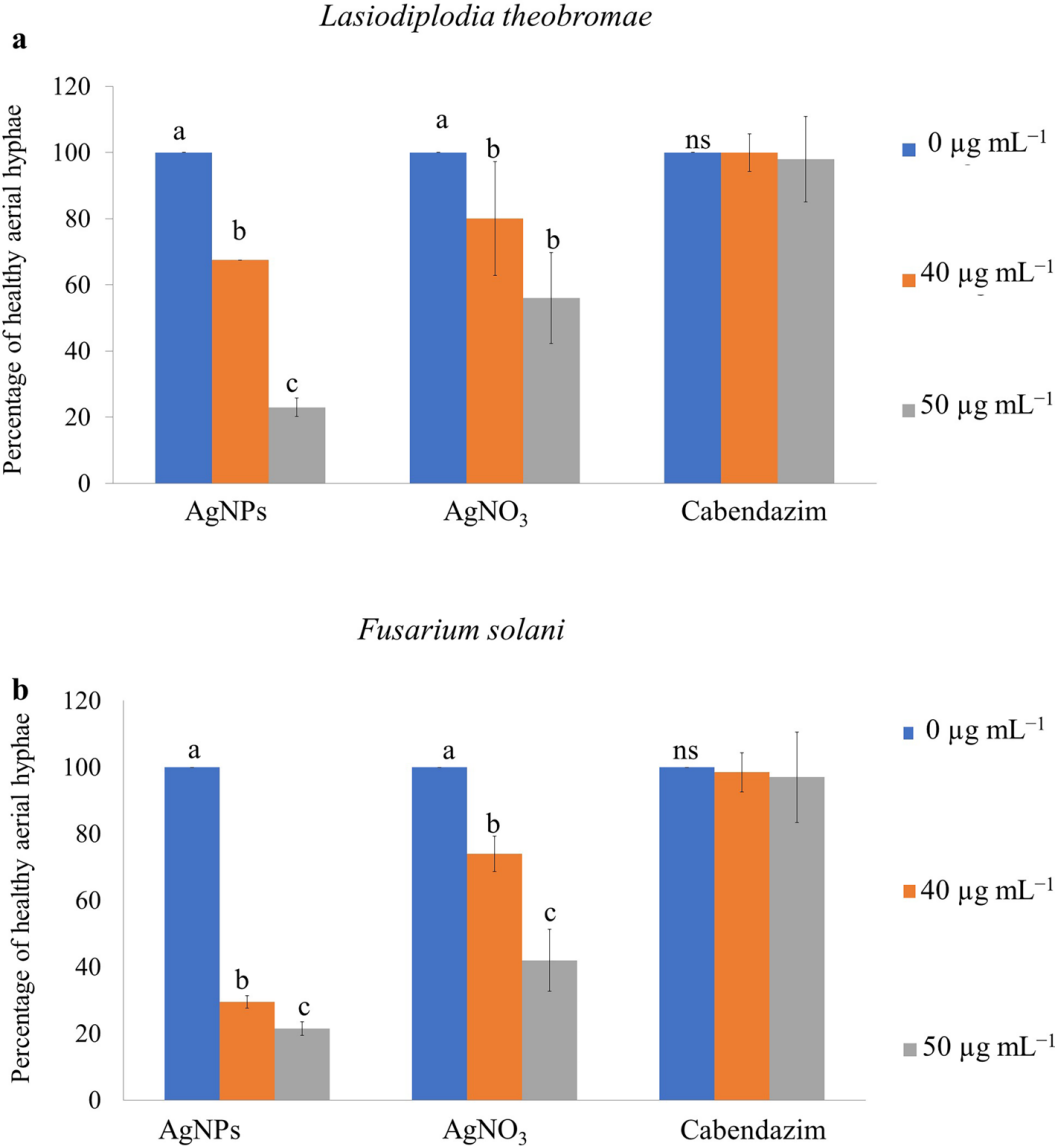
Quantification of healthy aerial hyphae on media containing AgNPs, AgNO_3_, and Carbendazim. (**a**) The healthy aerial hyphae rate of *Lasiodiplodia theobromae* and (**b**) the healthy aerial hyphae rate of *Fusarium solani*. Means in the graph followed by different letters is a are significantly different, ns = not statistically significant according to DMRT at P = 0.05.

To further test whether the development of *L. theobromae* or *F. solani* aerial hyphae growth was affected by AgNPs, additional experimentation was conducted under microscopy. The application of AgNPs caused a strong decrease in the percentage of healthy aerial hyphae of *L. theobromae* growth on the PDA plates, with only 67.50% by using 40 µg mL^−1^, and reduced to 23.00% by using 50 µg mL^−1^. Whereas the treatment of AgNO_3_ showed a decrease of healthy aerial hyphae to 80.00% and to 56.00% when using the previous respective concentrations. In the same trend, the percentage of healthy aerial hyphae of *F. solani* treated with AgNPs at 40 µg mL^−1^ and at 50 µg mL^−1^ decreased to 29.50 and 21.50% which resulted to be much more effective than using AgNO_3_ (74.00 and 42.00%). Moreover, the applied carbendazim at the same concentrations of AgNPs did not effectively inhibit hyphae growth as presented in Fig. 8.

### Biochemical changes of mycelium using SR-FTIR spectroscopy

**Figure 9:**
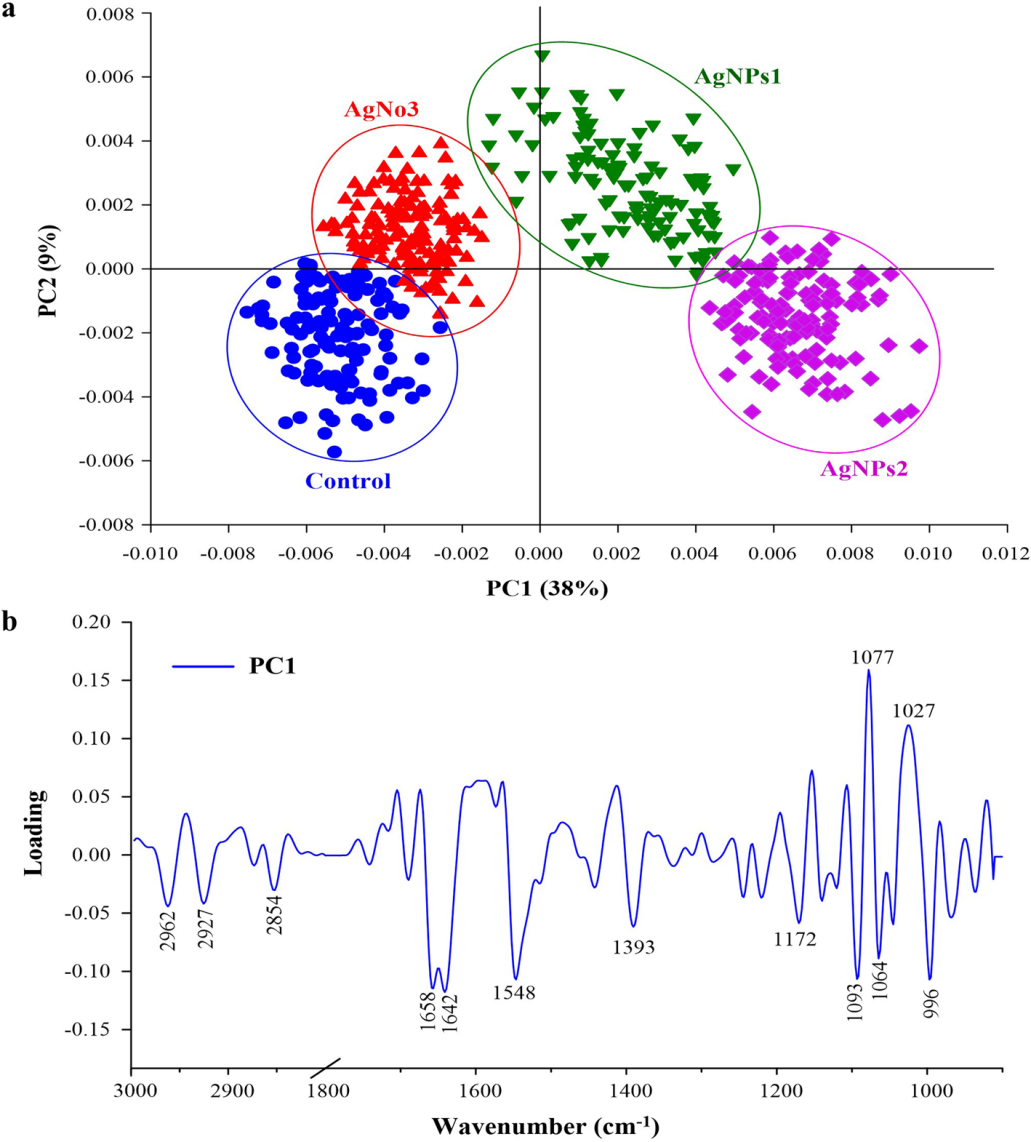
(**a**) Principle component analysis (PCA) of *Lasiodiplodia theobromae* and *Fusarium solani* treated with AgNPs1 (40 µg mL^−1^) and AgNPs2 (50 µg mL^−1^) in comparison to AgNO_3_ (50 µg mL^−1^) and untreated samples. (**b**) Loading plots from PCA analysis.

The SR-FTIR spectral data was analyzed to investigate biochemical changes in *L. theobromae* and *F. solani* mycelium after the application of the treatments with AgNPs and AgNO_3_. The results showed a clear separation of the chemical composition of mycelium between groups of the treated samples with 40 and 50 µg mL^−1^ of AgNPs in comparison to 50 µg mL^−1^ of AgNO_3_ and to untreated. These results are supported by the principal component analysis (PCA) score plot (Fig. 9a) which is explained by PC1 (38%) and PC2 (9%) that are linked to loading. The positive loading PC1 at 1077 and 1027 cm^−1^ was the one that significantly characterized the untreated samples which present higher intensity in the polysaccharide region. The highest negative loading from the PC1 at 2962, 2927, 2854, 1658, 1642 and 1548 cm^−1^ corresponded with the negative score plot obtained from the AgNPs treated samples which presented significant intensities in the protein and lipids region (Fig. 9b).

**Figure 10:**
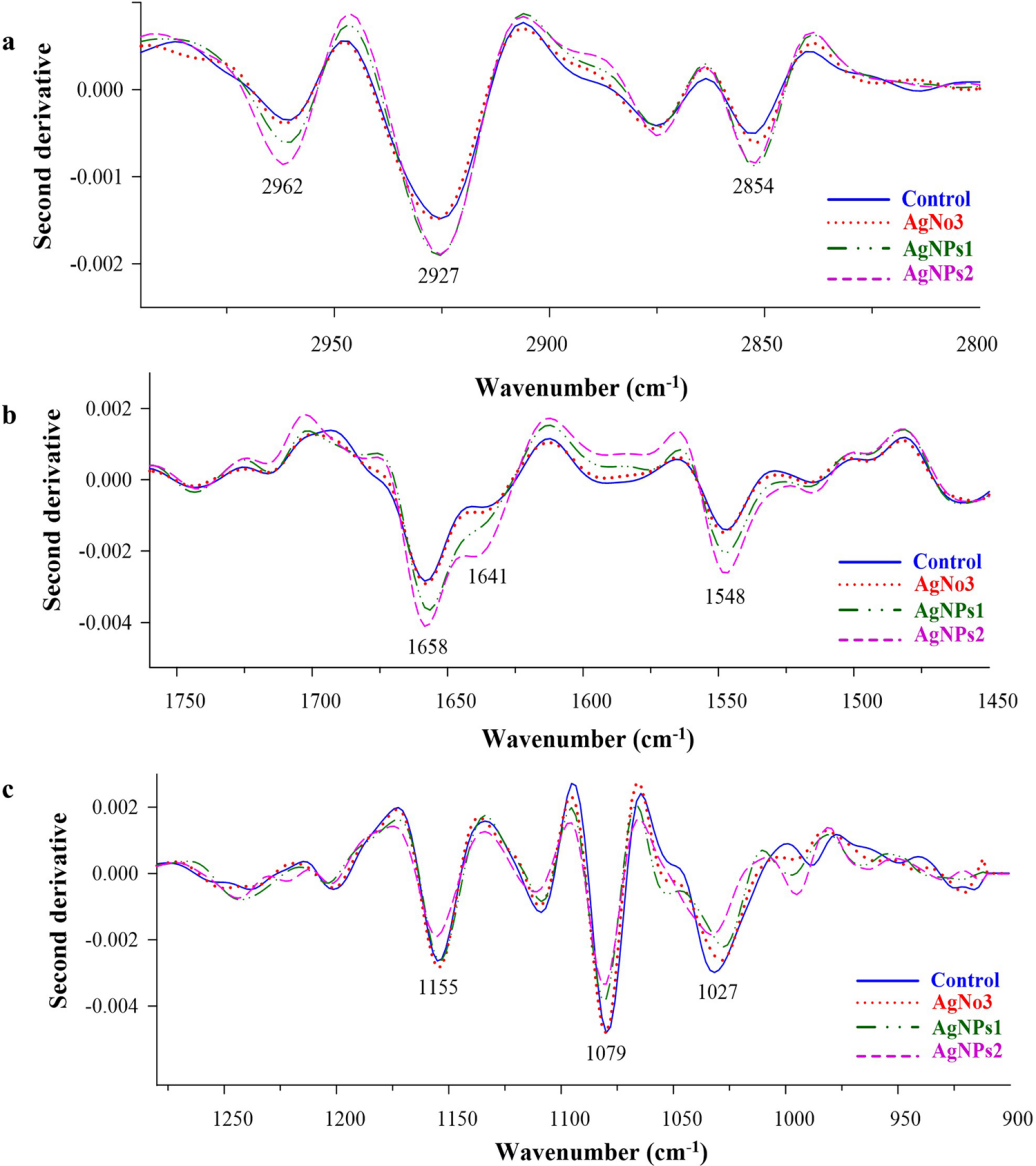
Average 2nd derivative spectrum of *L. theobromae* and *F. solani* treated with AgNPs1 (40 µg mL^−1^) and AgNPs2 (50 µg mL^−1^) in comparison to AgNO_3_ (50 µg mL^−1^) and untreated samples. (**a**) Region of 3000–2800 cm^−1^, (**b**) Region of 1700–1450 cm^−1^, and (**C**) Region of 1300–900 cm^−1^.

AgNPs treated samples showed differences in the spectra corresponding to the individual functional groups in three distinguishable regions (Fig. 10). The first region (3000–2800 cm^−1^) associated with mycelium composition which can be assigned to CH_2_ and CH_3_ stretch is mainly composed of lipids and fatty acids where the highest peaks were found at 2962, 2927, and 2854 cm^−1^ in the AgNPs treated samples. The second region (1700–1500 cm^−1^), composed by proteins and peptides with amide I and amide II, shows the highest peaks at 1658, 1642, and 1548 cm^−1^ in the AgNPs treated samples. The third region (1300–900 cm^−1^), which involves nucleic acid, phospholipids, polysaccharides, and carbohydrates, shows the highest peaks at 1155, 1079, and 1027 cm^−1^ from the untreated samples.

### Effect of bio-synthetized AgNPs to control cassava root rot disease

**Figure 11:**
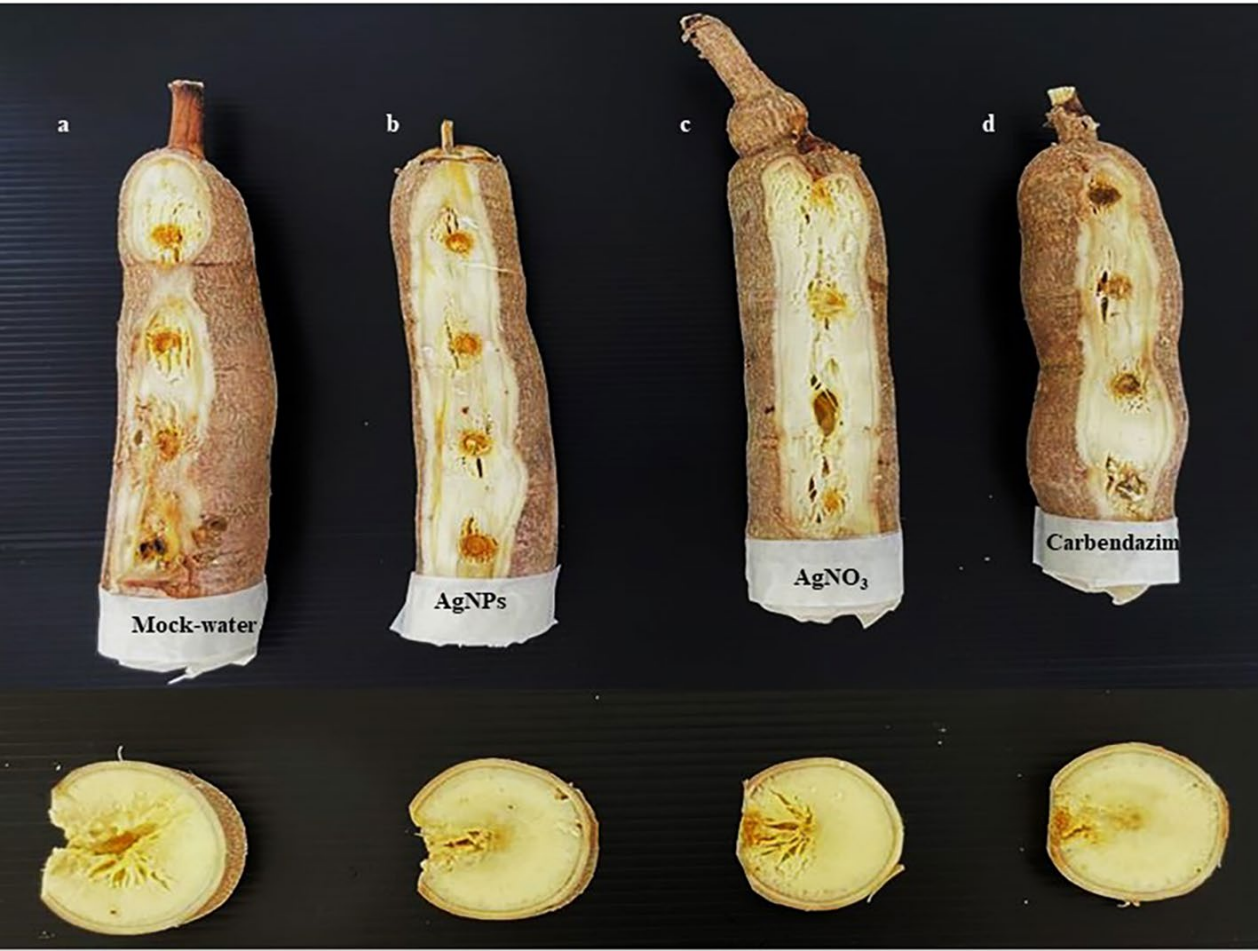
Comparison of different treatments of cassava (CMR 89) after being inoculated with *Lasiodiplodia theobromae* (**a**) Control, (**b**) AgNPs 50 µg mL^−1^, (**c**) AgNO_3_ 100 µg mL^−1^, and (**d**) Carbendazim 4 mg mL^−1^.

**Figure 12:**
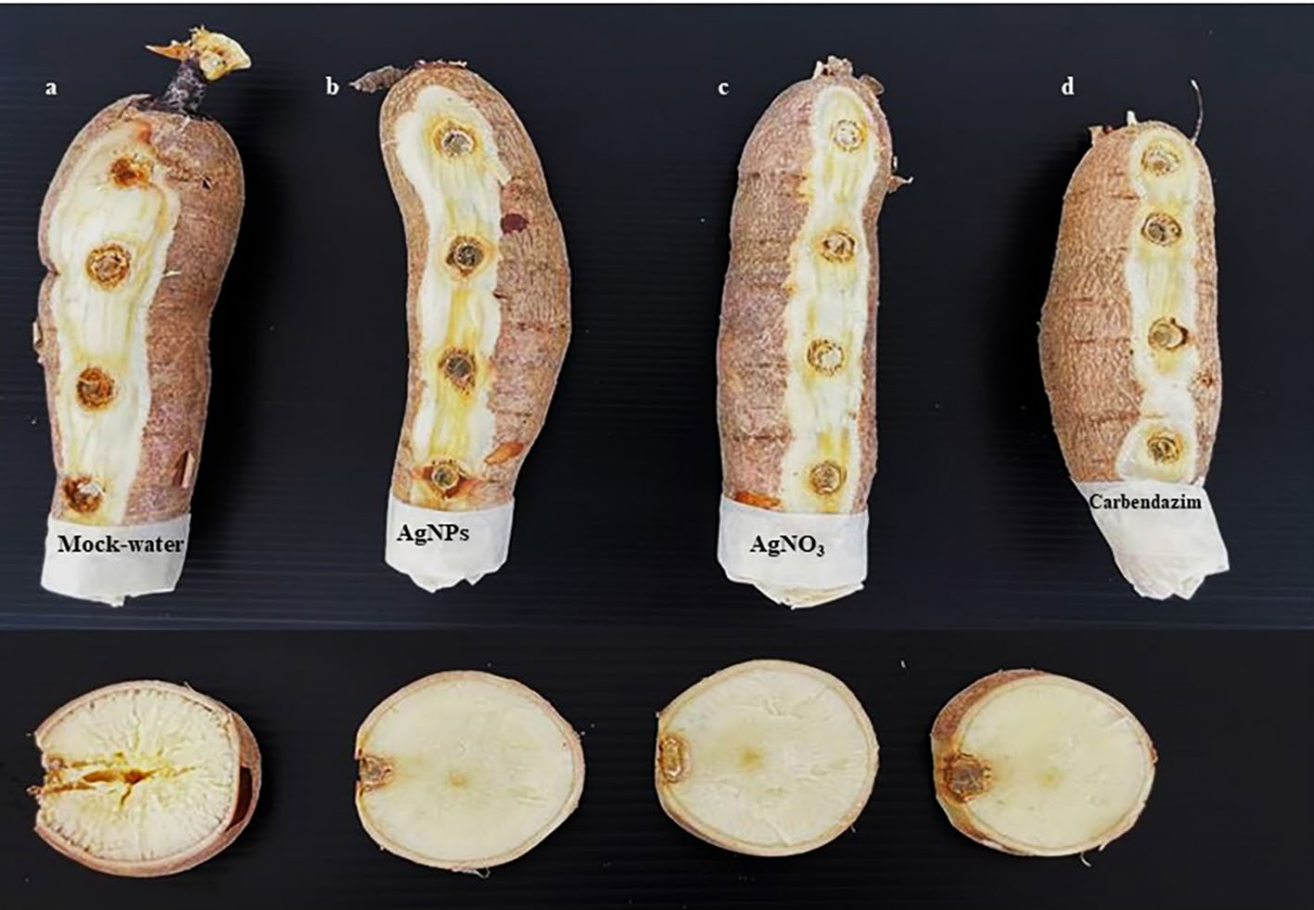
Comparison of different treatments of cassava (CMR 89) after being inoculated with *Fusarium solani* (**a**) Control, (**b**) AgNPs 50 µg mL^−1^, (**c**) AgNO_3_ 100 µg mL^−1^, and (**d**) Carbendazim 4 mg mL^−1^.

The potential effects of the biosynthesized AgNPs on reducing disease severity on cassava root cultivar CMR 89 were studied. The disease severity caused by *L. theobromae* in samples treated with 50 µg mL^−1^ of AgNPs (1.75 ± 0.50) was significantly lower than the severity observed from samples treated with 100 µg mL^−1^ of AgNO_3_ (2.50 ± 0.57) and from the control samples (5.00 ± 0.00). The AgNPs treatment had no significant different results compared to the 4 mg mL^−1^ carbendazim treatment (1.50 ± 0.57). Moreover, the AgNPs treatment also showed the lowest disease severity caused by *F. solani* as 1.00 ± 0.00 followed by carbendazim (1.25 ± 0.50) and AgNO_3_ (1.50 ± 0.57) which were all significantly effective compared to control (Table 3, Figs. 11 and 12).

**Table 3:** Effectiveness of biosynthesized silver nanoparticles on the disease severity of cassava root rot caused by *Lasiodiplodia theobromae* and *Fusarium solani.*

Treatment	Disease severity	Disease reduction (%)	Disease severity	Disease reduction (%)
	*Lasiodiplodia theobromae*		*Fusarium solani*	
Control	5.00 ± 0.00^a^	–	4.75 ± 0.50^a^	–
AgNPs	1.75 ± 0.50^c^	65.00 ± 10.00^a^	1.00 ± 0.00^b^	78.94 ± 0.00^a^
AgNO_3_	2.50 ± 0.57^b^	50.00 ± 11.54^b^	1.50 ± 0.57^b^	68.42 ± 10.31^a^
Carbendazim	1.50 ± 0.57^c^	70.00 ± 11.54^a^	1.25 ± 0.50^b^	73.69 ± 9.46^a^

The meaning of the different letters (a,b,c) indicated significant difference via DMRT at P = 0.05.

## Discussion

Bio-reduction of AgNO_3_ into AgNPs after the addition of *T. harzianum* supernatant was confirmed by its change in color. Initially, after the addition of the *T. harzianum* supernatant, the color turned to pale yellow and continued to change to light brown as the incubation time was increasing. After 24 h of incubation, the solution changes to deep brown color. A complete color change was seen at 48 h after incubation when no further color change was observed which indicated that all the AgNO_3_ was completely reduced to AgNPs. This change in color is an indication of the AgNP formation (reduction process) due to the excitation of the surface plasmon resonance (SPR) of silver nitrate^34^. Proteins, essential amino acids, and other substances present in *T. harzianum* supernatant serve as natural reducing and capping agents in the synthesis of AgNPs for reducing silver nitrate to AgNPs^35^. *T. harzianum* are filamentous fungi known for their ability to produce various enzymes, including reducing enzymes, that can catalyze the reduction of metal ions, such as Ag^+^ ions from silver nitrate, into their metallic nanoparticle form. The reducing enzymes present in the supernatant, such as nitrate reductase, act as catalysts to facilitate the reduction of Ag^+^ ions (silver cations) to metallic silver (Ag^0^). The enzymes provide electrons to the Ag^+^ ions, converting them into AgNPs. The DLS analysis measured the particle size and diffusion coefficient of the nanoparticles, confirming the synthesis of AgNPs using *T. harzianum* supernatant. The size of AgNPs using *T. harzianum* supernatant was smaller than using the other proposed treatments (Table 1) which may be caused by the capping effect of nanoparticles from the *T. harzianum* metabolites*.* The size of a nanoparticle influences its physiological reaction, distribution, and elimination. According to the DLS, the dispersion only contains nanoparticles with an average diameter size of 39.79 nm. The minimum particle size that can be measured by the DLS is smaller than the SEM which is convenient in this study (Fig. 3). This can be explained as the DLS considers the capping and the lowering agents when determining the size. It is important to note that, in addition to size, nanoparticle stability is critical for promoting biological activity^36^. The zeta potential quantifies the electrical charge on the surface of nanoparticles. Since higher zeta potential nanoparticles reject one another, they are less likely to form nano agglomerations and are more stable^37^. Particles with zeta potential greater than + 30 mV or less than − 30 mV are assumed to be more stable than those with intermediate zeta potentials^38^. The bio-synthesized AgNPs had a zeta potential of + 39.14 mV, indicating that they were highly stable. The electrostatic attraction of positively charged capping agents in nanoparticles caused the system to generate a positive zeta potential. The biological activity results showed that the AgNPs presented potential for the control of *L. theobromae* and *F. solani* since mycelium growth was reduced. The development of *L. theobromae* or *F. solani* aerial hyphae growth were affected by AgNPs-based treatment. Treatments using AgNO_3_, and carbendazim also resulted in the inhibition of *L. theobromae* and *F. solani* at higher concentrations as shown in Table 2; whereas, the AgNPs at lower concentrations can effectively reduce mycelium growth (Figs. 4 and 5). AgNPs are frequently used against crop diseases because of their antagonistic activity against a broad range of phytopathogens^22^. In this study, the bio-synthesized AgNPs showed inhibitory activity against hypha growth, remarking their potential application against root rot disease. For further understanding, an analysis on the biochemical changes of the pathogens mycelium using SR-FTIR microspectroscopy was performed. The lipid region was found at 2962, 2927, and 2854 cm^−1^ in the spectral data obtained from the AgNPs treated samples which principally provided asymmetric and symmetric stretching of CH_3_ and CH_2._ Lipids are the composition of fungus mycelium used to formulate edible coatings. Lipids are also the most abundant respiratory substrates in extra-radical mycelium, and they play critical roles in the fungus carbon metabolism and its transport^39^. Furthermore, lipid transporters in fungal membrane associated to biosynthetic and metabolic pathways contribute to virulence mechanisms that are important targets for protecting cell from environmental stress as well as for protein resistance, biofilm formation, and extracellular polysaccharide^40,41^. According to Walley et al.^42^, fatty acids also modulate a variety of signal transduction pathways triggered by environmental and developmental stimuli^42^. Our results are in accordance to the previous analysis conducted by Shapaval et al.^43^. The authors indicated that the fatty acid profile of the fungi *Mucor plumbeus* on different carbon source mediums led to changes in the FTIR spectra (3000–2800 cm^−1^) that revealed contents of unsaturated fatty acids. Similarly, Walley et al.^42^ reported that the increase in free fatty acid levels depends on stress responses which play a crucial role in pathogen membranes composition, and act as protected signaling molecules that is transported across eukaryotic cells for immune responses against biotic or abiotic stress. In addition, the membrane damage integrity mechanism contributes to deliver an increase of levels in the acyl chain, causing the peaks in the 3000–2800 cm^−1^ region to increase as well. The absorption band located at 1658, 1641, and 1548 cm^−1^ are related to the protein peptides; Amide I (1600–1690 C=O stretching vibration) and Amide II (1480–1575; CN and NH bending vibration) were assigned to alpha helical structures, lysine structure, and N-terminal amino group^44,45^. The group of amino acids, proteins, and polypeptide found in fungi are important for the well-functioning of their immune system, their adaptation to ecological niches or to severe environments, and for producing fungal immunomodulatory proteins (FIPs) and small secreted proteins (SSPs)^46,47^. In addition, fungi secrete diverse groups of amino acid groups through small proteins to be essential for their virulence^48^. Our results are in accordance with Skoneczny and Skoneczna^49^. The authors reported that eukaryotes, including yeast and fungi, can adjust their transcriptional response, allowing adaptation to various chemical and physical stresses by developing pathways of mitogen-activated protein kinase activation. Similarly, Künzler^50^ reported that the most important defense mechanism of fungi is a chemical enzyme based on secondary metabolites, peptides, and proteins which are usually stored and produced within the fungal cells. The third region, which references polysaccharides and carbohydrates of fungi mycelium, showed higher peaks at 1155, 1079, and 1027 cm^−1^ from spectral readings of the untreated samples. These results determined that the polysaccharides and carbohydrates as cell wall C–O–C shifted to lower lectures in the AgNPs treated samples. This observation demonstrates that the AgNPs causes damage to mycelium since there is a detected peak of spectra representing fungi cell membrane^51,52^. These suggestions agree with the report of Mihoubi et al.^51^. who observed DNA damages of yeast demonstrated by the intensity drop of peaks at 1048 cm^−1^ and 1079 cm^−1^ indicating a loss of nucleic acids^52^. Biochemical changes in mycelium can also be associated with the expression or production of pathogen virulence factors^52^. The virulence factor changes can affect the pathogen ability to infect, colonize, or evade the host immune responses^53^. Such changes lead to less virulent or less effective pathogens as well as reduce the production of enzymes or toxins that facilitate host penetration^54^, which can have a significant effect on samples with low disease severity levels.

AgNPs synthetized from *T. harzianum* act as an antifungal agent for controlling *L. theobromae* and *F. solani* mycelium germination. The effect of biosynthesized AgNPs was further examined for the control of the cassava root rot disease. The results showed that the efficacy of silver is greatly influenced by preventative applications of AgNPs which can have a better pre-penetration and colonization of fungi on cassava roots. As conclusion, this work demonstrated that *T. harzianum* can be used as a reducing agent to synthetize AgNPs for the inhibition of the germination of *L. theobromae* and *F. solani*, causal agents of cassava root rot disease.

## Data Availability

All data generated or analyzed during this study are included in this published article.

## References

[1] Siddiqi KS, Husen A, Rao RAK (2018). A review on biosynthesis of silver nanoparticles and their biocidal properties. J. Nanobiotechnol..

[2] Siddiqi KS, Husen A (2016). Fabrication of metal nanoparticles from fungi and metal salts: Scope and application. Nanoscale. Res. Lett..

[3] Raval N, Rakesh KT (2019). Basic Fundamentals of Drug Delivery.

[4] Nobbmann U (2007). Dynamic light scattering as a relative tool for assessing the molecular integrity and stability of monoclonal antibodies. Biotechnol. Genet. Eng. Rev..

[5] Vladár AE, Hodoroaba V-D, Hodoroaba VD, Unger WES, Shard AG (2020). Characterization of Nanoparticles by Scanning Electron Microscopy.

[6] Bootz A, Vogel V, Schubert D, Kreuter J (2004). Comparison of scanning electron microscopy, dynamic light scattering and analytical ultracentrifugation for the sizing of poly (Butyl cyanoacrylate) nanoparticles. Eur. J. Pharm. Biopharm..

[7] Ying S (2022). Green synthesis of nanoparticles: Current developments and limitations. Environ. Technol. Innov..

[8] Jeevanandam J, Barhoum A, Chan YS, Dufresne A, Danquah MK (2018). Review on nanoparticles and nanostructured materials: History, sources, toxicity and regulations. Beilstein J. Nanotechnol..

[9] Kumar A, Nagar S, Anand S, Singh P (2021). Plant-Microbes-Engineered Nano-particles (PM-ENPs) Nexus in Agro-Ecosystems: Understanding the Interaction of Plant, Microbes and Engineered Nano-particles (ENPS).

[10] Shang Y (2019). Applications of nanotechnology in plant growth and crop protection: A review. Molecules.

[11] Singh A, Prasad SM (2017). Nanotechnology and its role in agro-ecosystem: A strategic perspective. Int. J. Environ. Sci. Technol..

[12] Khan I (2022). Nanoparticle's uptake and translocation mechanisms in plants via seed priming, foliar treatment, and root exposure: A review. Environ. Sci. Pollut. Res. Int..

[13] Saravanan A (2021). A review on biosynthesis of metal nanoparticles and its environmental applications. Chemosphere.

[14] Iravani S, Korbekandi H, Mirmohammadi SV, Zolfaghari B (2014). Synthesis of silver nanoparticles: chemical, physical and biological methods. Res. Pharm. Sci..

[15] Arzu Ö, Dilek A, Muhsin K, Larramendy LM, Soloneski S (2016). Environmental Health Risk, Ch. 1.

[16] Fariq A, Khan T, Yasmin A (2017). Microbial synthesis of nanoparticles and their potential applications in biomedicine. J. Appl. Biomed..

[17] Rozhin A (2021). Biogenic silver nanoparticles: Synthesis and application as antibacterial and antifungal agents. Micromachines.

[18] Guilger M (2017). Biogenic silver nanoparticles based on *Trichoderma harzianum*: synthesis, characterization, toxicity evaluation and biological activity. Sci. Rep..

[19] Dorcheh SK, Vahabi K, Mérillon JM, Ramawat KG (2017). Fungal Metabolites.

[20] Contreras-Cornejo HA, Macías-Rodríguez L, del Val E, Larsen J (2016). Ecological functions of *Trichoderma* spp. and their secondary metabolites in the rhizosphere: Interactions with plants. FEMS Microbiol. Ecol..

[21] Saengchan C, Phansak P, Le Thanh T, Papathoti NK, Buensanteai N (2021). Efficacy of salicylic acid and a *Bacillus* bioproduct in enhancing growth of cassava and controlling root rot disease. J. Plant Prot. Res..

[22] Kim SW (2012). Antifungal effects of silver nanoparticles (AgNPs) against various plant pathogenic fungi. Mycobiology.

[23] Oliveira DGP, Pauli G, Mascarin GM, Delalibera I (2015). A protocol for determination of conidial viability of the fungal entomopathogens *Beauveria bassiana* and *Metarhizium anisopliae* from commercial products. J. Microbiol. Methods.

[24] Alsamhary KI (2020). Eco-friendly synthesis of silver nanoparticles by *Bacillus*
*subtilis* and their antibacterial activity. Saudi J. Biol. Sci..

[25] Madakka M, Jayaraju N, Rajesh N (2018). Mycosynthesis of silver nanoparticles and their characterization. MethodsX.

[26] Carvalho PM, Felício MR, Santos NC, Gonçalves S, Domingues MM (2018). Application of light scattering techniques to nanoparticle characterization and development. Front. Chem..

[27] Delvallée A, Feltin N, Ducourtieux S, Trabelsi M, Hochepied JF (2015). Direct comparison of AFM and SEM measurements on the same set of nanoparticles. Meas. Sci. Technol..

[28] Balouiri M, Sadiki M, Ibnsouda SK (2016). Methods for in vitro evaluating antimicrobial activity. J. Pharm. Anal..

[29] Makovitzki A, Viterbo A, Brotman Y, Chet I, Shai Y (2007). Inhibition of fungal and bacterial plant pathogens in vitro and in planta with ultrashort cationic lipopeptides. Appl. Environ. Microbiol..

[30] John HR (2008). Reference method for broth dilution antifungal susceptibility testing of filamentous fungi, approved standard. Second edition. M38–A2. Clin. Lab. Stand. Inst..

[31] Kogkaki E (2017). Differentiation and identification of grape-associated black aspergilli using Fourier transform infrared (FT-IR) spectroscopic analysis of mycelia. Int. J. Food Microbiol..

[32] Onyeka TJ, Dixon AG, Ekpo EJ (2005). Assessment of laboratory methods for evaluating cassava genotypes for resistance to root rot disease. Mycopathologia.

[33] Boas S, Hohenfeld C, Oliveira S, Santos V, Oliveira E (2016). Sources of resistance to cassava root rot caused by *Fusarium* spp.: A genotypic approach. Euphytica.

[34] Mahmudin L, Suharyadi E, Utomo A, Abraha K (2015). Optical properties of silver nanoparticles for surface plasmon resonance (SPR)-based biosensor applications. J. Mod. Phys..

[35] Konappa N (2021). Ameliorated antibacterial and antioxidant properties by *Trichoderma harzianum* mediated green synthesis of silver nanoparticles. Biomolecules.

[36] McNamara, K., Tofail, S. A. M., Thorat, N. D., Bauer, J. & Mulvihill, J. J. E. (2020) In: Florent, C. (eds) *Nanoalloys, 2nd edn.* Elsevier, Paris, pp 381–432

[37] Shnoudeh AJ, Tekade RK (2019). Synthesis, characterization, and applications of metal nanoparticles. Biomaterials and Biotechnology.

[38] Clogston J, Patri A (2011). Zeta potential measurement. Methods Mol. Biol..

[39] Van Aarle IM, Olsson PA (2003). Fungal lipid accumulation and development of mycelial structures by two arbuscular mycorrhizal fungi. Appl. Environ. Microbiol..

[40] Rella A, Farnoud AM, Del Poeta M (2016). Plasma membrane lipids and their role in fungal virulence. Prog. Lipid Res..

[41] Salvatore MM, Alves A, Andolfi A (2020). Secondary metabolites of *Lasiodiplodia theobromae*: Distribution, chemical diversity, bioactivity, and implications of their occurrence. Toxins.

[42] Walley JW, Kliebenstein DJ, Bostock RM, Dehesh K (2013). Fatty acids and early detection of pathogens. Curr. Opin. Plant. Biol..

[43] Shapaval V, Afseth NK, Vogt G, Kohler A (2014). Fourier transform infrared spectroscopy for the prediction of fatty acid profiles in mucor fungi grown in media with different carbon sources. Microb. Cell Fact..

[44] Krimm S, Bandekar J, Anfinsen CB, Edsall JT, Richards FM (1986). Advances in Protein Chemistry.

[45] Bandekar J (1992). Amide modes and protein conformation. Biochim. Biophys. Acta Protein Struct. Mol. Enzymol..

[46] Feldman D, Yarden O, Hadar Y (2020). Seeking the roles for fungal small-secreted proteins in affecting saprophytic lifestyles. Front. Microbiol..

[47] Liu Y, Bastiaan-Net S, Wichers HJ (2020). Current understanding of the structure and function of fungal immunomodulatory proteins. Front. Nutr..

[48] Kämper J (2006). Insights from the genome of the biotrophic fungal plant pathogen *Ustilago*
*maydis*. Nature.

[49] Skoneczny M, Skoneczna A, Skoneczny M (2018). Stress Response Mechanisms in Fungi: Theoretical and Practical Aspects.

[50] Künzler M (2018). How fungi defend themselves against microbial competitors and animal predators. PLoS Pathog..

[51] Mihoubi W, Sahli E, Gargouri A, Amiel C (2017). FTIR spectroscopy of whole cells for the monitoring of yeast apoptosis mediated by p53 over-expression and its suppression by *Nigella*
*sativa* extracts. PLoS One.

[52] Peyraud R, Mbengue M, Barbacci A, Raffaele S (2019). Intercellular cooperation in a fungal plant pathogen facilitates host colonization. Proc. Natl. Acad. Sci. USA.

[53] Brown AJP, Cowen LE, di Pietro A, Quinn J (2017). The Fungal Kingdom.

[54] Walter S, Nicholson P, Doohan FM (2010). Action and reaction of host and pathogen during Fusarium head blight disease. New Phytol..

